# Rapid fiber-detection technique by artificial intelligence in phase-contrast microscope images of simulated atmospheric samples

**DOI:** 10.1093/annweh/wxae014

**Published:** 2024-03-04

**Authors:** Takashi Yamamoto, Kazuharu Iwasaki, Yukiko Iida, Ken-ichi Yuki, Fumihiro Nakaji, Hayato Yamashiro, Toshiyuki Toyoguchi, Atsushi Terazono

**Affiliations:** National Institute for Environmental Studies, 16-2 Onogawa, Tsukuba, Ibaraki 305-8506, Japan; Japan NUS Co., Ltd, 7-5-25 Nishi-Shinjuku, Shinjuku, Tokyo 160-0023, Japan; Environmental Control Center Co., Ltd, 3-7-23 Sanda-machi, Hachioji, Tokyo 193-0832, Japan; Environmental Control Center Co., Ltd, 3-7-23 Sanda-machi, Hachioji, Tokyo 193-0832, Japan; Japan NUS Co., Ltd, 7-5-25 Nishi-Shinjuku, Shinjuku, Tokyo 160-0023, Japan; Japan NUS Co., Ltd, 7-5-25 Nishi-Shinjuku, Shinjuku, Tokyo 160-0023, Japan; Environmental Control Center Co., Ltd, 3-7-23 Sanda-machi, Hachioji, Tokyo 193-0832, Japan; National Institute for Environmental Studies, 16-2 Onogawa, Tsukuba, Ibaraki 305-8506, Japan

**Keywords:** artificial intelligence, asbestos, phase-contrast microscopy, rapid detection

## Abstract

Since the manufacture, import, and use of asbestos products have been completely abolished in Japan, the main cause of asbestos emissions into the atmosphere is the demolition and removal of buildings built with asbestos-containing materials. To detect and correct asbestos emissions from inappropriate demolition and removal operations at an early stage, a rapid method to measure atmospheric asbestos fibers is required. The current rapid measurement method is a combination of short-term atmospheric sampling and phase-contrast microscopy counting. However, visual counting takes a considerable amount of time and is not sufficiently fast. Using artificial intelligence (AI) to analyze microscope images to detect fibers may greatly reduce the time required for counting. Therefore, in this study, we investigated the use of AI image analysis for detecting fibers in phase-contrast microscope images. A series of simulated atmospheric samples prepared from standard samples of amosite and chrysotile were observed using a phase-contrast microscope. Images were captured, and training datasets were created from the counting results of expert analysts. We adopted 2 types of AI models—an instance segmentation model, namely the mask region-based convolutional neural network (Mask R-CNN), and a semantic segmentation model, namely the multi-level aggregation network (MA-Net)—that were trained to detect asbestos fibers. The accuracy of fiber detection achieved with the Mask R-CNN model was 57% for recall and 46% for precision, whereas the accuracy achieved with the MA-Net model was 95% for recall and 91% for precision. Therefore, satisfactory results were obtained with the MA-Net model. The time required for fiber detection was less than 1 s per image in both AI models, which was faster than the time required for counting by an expert analyst.

**What’s important about this paper**

This study demonstrated a method for rapidly and accurately detecting fibers using artificial intelligence models to analyze phase-contrast microscope images of simulated atmospheric samples prepared from amosite and chrysotile standard samples. The method can be useful for the early detection and prevention of asbestos fiber emissions during the demolition and removal of buildings that use asbestos-containing building materials.

## Introduction

Asbestos is a group of natural fibrous silicate minerals with excellent properties, such as high tensile strength, heat insulation, fire resistance, and sound insulation. In the past, large amounts of asbestos were used to produce building materials because it was available in large quantities and inexpensive. However, inhalation of asbestos causes fatal diseases such as lung cancer and mesothelioma (World Health Organization 1986; Dodson and Hammar 2006; Morinaga 2008). In Japan, asbestos usage peaked in the period from the 1970s to the 1990s. Although several decades have since passed, the annual mesothelioma death rate in Japan exceeded 1,500 people in 2022 (Ministry of Health, Labour and Welfare of Japan 2022).

Due to several health hazard incidences, the use of asbestos was gradually regulated in Japan. The manufacture, import, and use of products with asbestos concentrations exceeding 0.1% by weight was banned in 2006 (Ministry of Health, Labour and Welfare of Japan 2006). Asbestos was mainly used to manufacture asbestos-containing building materials (ACBMs), such as sprayed asbestos, asbestos-containing insulation materials, and asbestos-cement slate boards. Although many buildings constructed with ACBMs are still in use today, they will be demolished at the end of their lifespans. According to the Ministry of Land, Infrastructure, Transport, and Tourism of Japan (2009), the number of private buildings constructed with ACBMs was approximately 2.8 million in 2009, and the number of buildings demolished is expected to increase until 2028 and reach a peak of approximately 100,000 buildings per year.

Since the manufacture and use of asbestos are currently prohibited in Japan, the demolition and removal of buildings with ACBMs are some of the causes of atmospheric asbestos emissions. Maintenance works or damage of ACBMs may also cause asbestos emissions. Additionally, damage to buildings caused by disasters such as earthquakes can also cause asbestos emissions (Terazono et al. 1999). In Japan, the Air Pollution Control Law stipulates working standards to prevent the emission of asbestos during demolition and removal work; however, asbestos emission incidents often occur because of inappropriate operations. An atmospheric asbestos environmental survey conducted by the Ministry of the Environment at the security zone entrance/exit of a demolition site found a concentration of asbestos fibers in the sampled atmosphere of 54 fibers/L (Ministry of the Environment of Japan 2022a).

To detect and correct asbestos emissions at an early stage, a rapid measurement method for atmospheric asbestos fibers is required. The current measurement method stipulated by the Ministry of the Environment is a combination of air sampling in a short time of 30 min or less, increasing the suction flow rate within a range of 5–20 L/min, and counting fibers in 100 (or 50) fields of view using phase-contrast microscope (PCM) images (Ministry of the Environment of Japan 2022b). The time for counting 100 fields of view depends on the fiber density of the filter and the ability of the analyst, but it takes 25 to 100 min, a speed that is insufficient for correcting asbestos emissions quickly. In addition, there is a possibility that fibers other than asbestos are scattered at demolition and removal sites. With the increasing number of buildings to be demolished, monitoring of demolition scatter is also expected to increase. The limited number of expert analysts and the amount of time it takes to train them have resulted in a strong demand for labor-saving and automated fiber counting.

Attempts to automate the process of detecting and counting asbestos fibers in microscope images began in the 1980s (Kenny 1984; Baron and Shulman 1987; Inoue et al. 1998). At first, this was based on simple image analysis; however, since the 2000s, remarkable developments have taken place in artificial intelligence (AI), such as machine learning and deep learning. These developments have increased the number of cases in which AI has been applied to detect asbestos fibers in images (Moriguchi et al. 2009; Biswas and Biswas, 2021; Cai et al. 2021; Iida et al. 2021). Moriguchi et al. (2009) used a support vector machine to detect local color features in the asbestos analysis of building materials using the dispersion staining method. Biswas and Biswas (2021) applied a semantic segmentation model (U-Net) to images captured using a scanning electron microscope; of the 100 images that included amosite fibers, the AI model was trained using 80 images as training datasets and 20 images as evaluation datasets. As a result, the model was able to detect amosite fibers with 95% accuracy. Cai et al. (2021) applied a YOLOv4 model, which is an object detection convolutional neural network (CNN) model, to images captured with a fluorescence microscope and reported that they achieved a detection accuracy of 96.1%. Iida et al. (2021) compared the fiber counting results of 108 scanning electron microscope images obtained by an experienced operator and an AI model. The false-negative rate of the AI model compared to the counting results of the experienced operator was 12.1%, and the false-positive rate was 21.4%. The time required for counting was 150 min for the experienced operator and 3 min for the AI model, indicating a significant reduction in the time required. Thus, using AI for detecting fibers in microscope images may enable asbestos fibers to be measured in the atmosphere quickly and with an accuracy that equals that of expert analysts. Recent studies have focused on images obtained by scanning electron microscopy or fluorescence microscopy, and few have used PCM images, which are commonly used in Japan and other countries. Moreover, an object detection model, such as the YOLOv4 model, cannot recognize the shape of the detected object or determine whether the detected fiber satisfies the counting criteria. Therefore, it is uncertain whether the AI models employed in these studies are appropriate when considering the acceleration and automation of fiber detection in PCM images.

Although the measurement time is not dramatically reduced due to a reasonable amount of time required for atmospheric sampling, preparation of slides for observation, microscope setup, and checking the results, at least the time required for counting fibers under a PCM can be reduced by using AI to detect fibers. Moreover, according to “HSG248: Asbestos: Analyst’s Guide” (Health and Safety Executive 2021), fiber counting performed by analysts is affected by human error and fatigue. In particular, regarding fatigue, an upper limit on the number of fields of view that an analyst can count per task is stipulated as 2,400 fields of view in the “Human factors” section, since fatigue can impair the quality of fiber counting. The introduction of AI has the potential to circumvent these constraints on analysts. Analysts also generally tend to undercount fibers in samples with high dust density and overcount fibers in samples with low dust density (World Health Organization 1997), as well as have uncertainty in discriminating fiber size. It is unlikely that these problems will occur in fiber detection by AI. On the other hand, however, fiber detection by AI may be greatly affected by the quality of microscopic images.

Therefore, to develop a technique that can be applied to the rapid measurement of asbestos fibers in the atmosphere, we evaluated 2 AI models, an instance segmentation model, namely the mask region-based convolutional neural network (Mask R-CNN) (He et al. 2018), and a semantic segmentation model, namely the multi-level aggregation network (MA-Net) (Fan et al. 2020), for detecting fibers in PCM images of simulated atmospheric samples.

## Methods

### Preparation of simulated atmospheric samples and acquisition of images

To simulate atmospheric air samples, membrane filters were prepared by filtering asbestos standard samples dispersed in water. An appropriate amount of chrysotile standard sample (Union for International Cancer Control, Geneva, Switzerland) or amosite standard sample (The Japan Association for Working Environment Measurement, Tokyo, Japan) was dispersed in isopropyl alcohol and diluted with purified water. A portion of this diluted sample was separated and subjected to suction filtration through a mixed cellulose ester membrane filter (47 mmφ, 0.8 µm pore size). The filter was dried in a desiccator, cut to an appropriate size, placed on a glass slide, and cleared with acetone vapor. Triacetin was added dropwise to the cleared filter for fixation, and a coverslip was put on it. The coverslip has 50 circular fields of view, 300 µm in diameter, arranged in 5 rows (1 to 5) and 10 columns (A to J) (WESST Co., Ltd., Kanagawa, Japan) (Supplementary Fig. S1). The observed field of view was specified by the row and column numbers (e.g. A1). Five observation slides were prepared for each chrysotile (Chr-1 to Chr-5) and amosite (Amo-1 to Amo-5) standard sample. The observation slides were observed at 400× magnification using an Olympus BX53 PCM (Evident Corp., Tokyo, Japan) equipped with an Olympus UPLFLN40XPH objective lens and an Olympus WH10X eyepiece lens. PCM images were captured with an Olympus DP73 digital camera attached to the PCM via a C-mount adapter (Olympus U-TV0.35XC-2) in JPEG format (1,600 × 1,200 pixels). For each chrysotile and amosite slide, an expert analyst manually adjusted the focus and captured images of 20 fields of view. Simultaneously, the expert analyst counted fibers according to the fiber counting criteria of the “Asbestos Monitoring Manual” (Ministry of the Environment of Japan 2022b), and the reference counts were determined from the counting results; these criteria are fiber length of ≥5 µm, fiber width of <3 µm, and aspect ratio of ≥3. An expert analyst is a person who has passed the most advanced rank A of the proficiency test for asbestos fiber counting in Japan. Using an annotation tool (LabelMe; Russell et al. 2008), we manually extracted the area occupied by the fibers in the PCM images that were targeted for counting by the expert analyst and created training datasets (Supplementary Fig. S2).

Microscale images were viewed and photographed under the same conditions to obtain the length per pixel. A length of 10 µm equaled 32 pixels in the image; thus, this was set to 0.3125 µm/pixel.

### Fiber detection in images using AI models

As the AI models used for detecting fibers in images, we adopted the Mask R-CNN (He et al. 2018), an instance segmentation model, and MA-Net (Fan et al. 2020), a semantic segmentation model.

Atmospheric samples collected at demolition and removal work sites are assumed to have multiple fibers entangled or particles attached to fibers, making it difficult to detect the fibers. Thus, we adopted the Mask R-CNN model with the expectation that fibers could be detected and differentiated from other fibers and particles, even in such cases. The Mask R-CNN model consisted of a backbone that extracted image features by multiple convolution processes, a region proposal network that narrowed down the object candidate region (region of interest) from the feature map extracted by the backbone, and a head that performed object detection (present rectangle enclosing the object), object classification (calculation of classification probability), and outputted the segmentation mask. Thus, this model simultaneously performed 3 tasks: (i) object detection, (ii) object classification, and (iii) segmentation mask output. The segmentation mask is an image (mask) created by binary classification of pixels in the original image, with the values of pixels that constitute the detected object set as 1 and the values of other pixels set as 0. In this study, the Mask R-CNN model outputted 3 items: (i) the fiber position in the image, (ii) the fiber class probability, and (iii) the segmentation mask. Results with a fiber class probability of more than 50% were extracted, and a group of pixels with a segmentation mask value of 0.8 or more was defined as a fiber. A rectangle circumscribing this outline was obtained through image analysis. The number of pixels on the long side was taken as the fiber length, and the number of pixels orthogonal to the long side at the center point of the detected fiber was taken as the fiber width (Supplementary Fig. S3). Fibers that satisfied the aforementioned fiber counting criteria were counted.

The semantic segmentation model, similar to the instance segmentation model, performed object classification at the pixel level; therefore, it was a model that could recognize the shape of fibers. The MA-Net adopted in this study was an extended u-net model used by Biswas and Biswas (2011). Two blocks were added to the u-net: the position-wise attention block and the multi-scale fusion attention block. The position-wise attention block captured spatial dependencies between pixels in feature maps, which is difficult to achieve with a CNN alone. The multi-scale fusion attention block captured channel dependencies. The MA-Net model outputted a binary classification probability for each pixel as fiber or background. A group of pixels with a probability greater than 50% was extracted as a fiber, and a rectangle that circumscribed the outline and rotated was obtained. The pixels extracted as fibers on the short side of the rectangle were counted, and the average value was considered as the fiber width.

### Learning procedure of the AI models

**Fig. 1.**
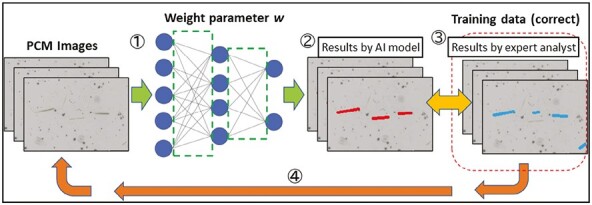
Schematic of the learning procedure used for the Mask R-CNN model. AI, artificial intelligence; Mask R-CNN, mask region-based convolutional neural network; PCM, phase-contrast microscope.

Of the 40 sets of image and training datasets, 30 were used to train the models and 10 were used to evaluate the results of the models’ fiber detection. In the learning model, we used a stochastic gradient descent with momentum method. A schematic of the learning procedure is shown in Fig. 1. The scheme was as follows: (i) image data were inputted for learning and detection of fibers; (ii) the error between the detection result of the fibers and the correct training data was calculated; (iii) the gradient for each weight parameter, *w*, was calculated, and the weight parameter between nodes was set; and (iv) the weight parameters were updated and repeated to approach the correct result. No image processing or manipulation was performed prior to AI processing.

The time required to train the model was approximately 30 min using both the CPU and graphic processing unit (GPU). Supplementary Table S1 shows the specifications of the computer used for learning, Supplementary Table S2 shows the library used, and Supplementary Table S3 lists the parameters used during learning.

### Evaluation of the models’ accuracies in detecting fibers

The AI models were judged to have correctly identified a fiber when the intersection over union ratio between the fiber predicted by the model and the evaluation data was ≥0.5. The intersection over union ratio value was obtained by dividing the area value of the overlapping portion of the fiber pixels of the evaluation data and the fiber pixels predicted by the AI model by the sum of their area values (Supplementary Fig. S4).

The accuracies of the AI models were evaluated using 2 indices, recall and precision. Recall is the percentage of evaluation data that can be predicted by the model, and precision is the percentage of correct predictions when compared with the reference count determined by the expert analyst. These are calculated by the following equations:


$$\begin{array}{l} Recall\;=\;True\;positive/\left(True\;positive\;+\;False\;negative\right) \end{array}$$ (1)

$$\begin{array}{l} Precision\;=\;True\;positive/\left(True\;positive\;+\;False\;positive\right) \end{array}$$ (2)

where,

True Positive: the number of fibers detected by the AI model that were counted by the expert analyst.

False Negative: the number of fibers counted by the expert analyst that the AI model did not detect (overlooked).

False Positive: the number of fibers detected by the AI model that were not counted by the expert analyst (overcounted).

## Results and Discussion

Table 1 summarizes the results of the fiber counting by the expert analyst and the fiber detection by the 2 models. Each image showed an average of 5.5 fibers per field of view, corresponding to an airborne fiber concentration of 31.2 fibers/L when assuming that 2,400 L of air is collected using a membrane filter with a diameter of 47 mm.

**Table 1.** The fiber detection results of the Mask R-CNN and MA-Net models for simulated atmospheric sample images.

Images	Counts by expert analyst	Artificial intelligence model (Mask R-CNN)			Artificial intelligence model (MA-Net)		
		Counted	Overlooked	Overcounted	Counted	Overlooked	Overcounted
Amo-1-B1	2	4	0	2	3	0	1
Amo-2-C4	5	5	1	1	6	0	1
Amo-3-B5	4	5	0	1	4	0	0
Amo-4-H3	5	4	3	2	6	0	1
Amo-5-A4	9	6	3	0	9	0	0
Chr-1-D2	5	1	4	0	5	0	0
Chr-3-B5	5	3	2	0	6	0	1
Chr-4-A2	9	7	2	0	8	1	0
Chr-5-E3	3	2	2	1	4	0	1
Chr-5-E4	8	4	4	0	6	2	0
Total	55	41	21	7	57	3	5

Amo, amosite; Chr, chrysotile; MA-Net, multi-level aggregation network; Mask R-CNN, mask region-based convolutional neural network.

**Fig. 2.**
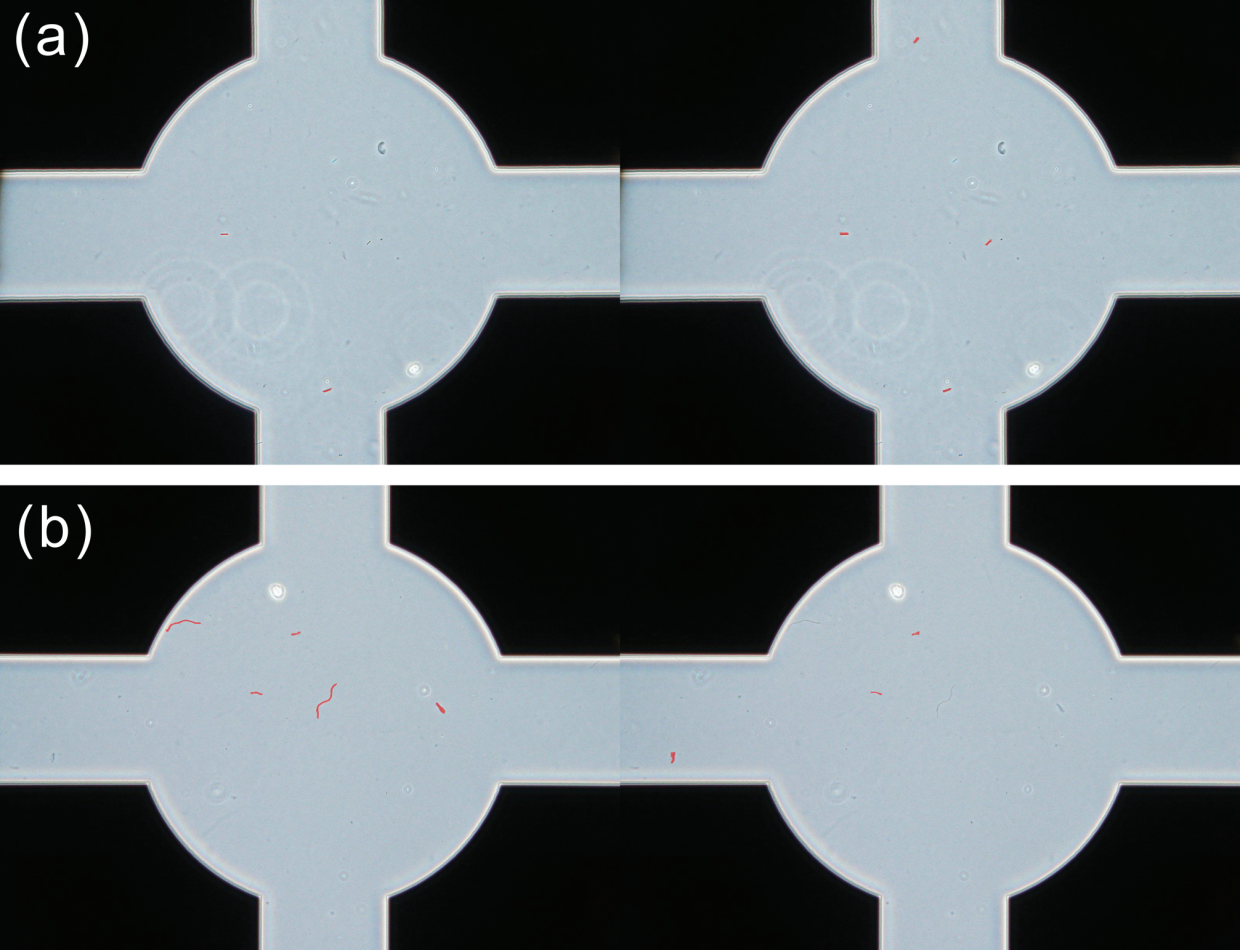
The Mask R-CNN model’s fiber detection results for (a) amosite (Amo-1-B1) and (b) chrysotile (Chr-1-D2) slide images. The fiber counting results by the expert analyst are shown on the left, and the fiber detection results by the AI model are shown on the right. Mask R-CNN, mask region-based convolutional neural network.

Figure 2 shows an example of the fiber detection results obtained using the Mask R-CNN model. Of the 55 fibers in the evaluation dataset counted by the expert analyst, 34 were detected as fibers to be counted, 21 were overlooked, and 7 were overcounted. The Mask R-CNN model tended to overcount the fibers in the amosite slide images (Fig. 2a); this was because the model detected fibers shorter than 5 µm, which the analyst did not count. However, the Mask R-CNN model tended to overlook fibers in the chrysotile slide images; in particular, many wavy fibers, such as those in the center of Fig. 2b, could not be detected.

**Fig. 3.**
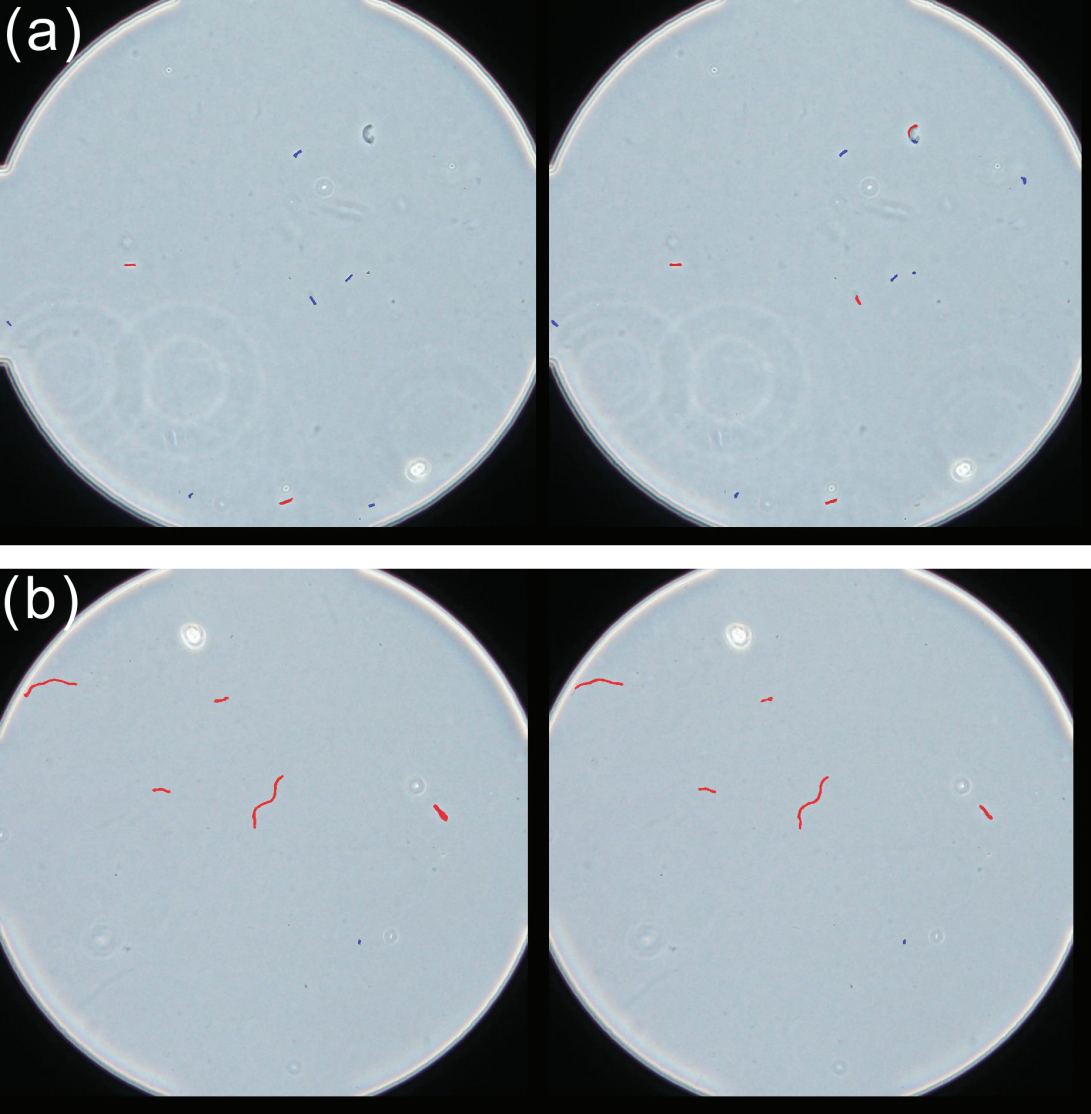
The MA-Net model’s fiber detection results for (a) amosite (Amo-1-B1) and (b) chrysotile (Chr-1-D2) slide images. The fiber counting results by the expert analyst are shown on the left, and the fiber detection results by the AI model are shown on the right. Fibers highlighted in red are fibers that were determined by the expert analyst or the AI model to have met the fiber counting criteria, and those in blue did not meet the criteria. MA-Net, multi-level aggregation network.

Figure 3 shows an example of the fiber detection results obtained using the MA-Net model. Of the 55 fibers in the evaluation dataset, 52 were detected as fibers to be counted, 3 were overlooked, and 5 were overcounted. The 3 fibers that were overlooked were detected as fibers, but the model determined that they did not meet the fiber count criteria. Wavy fibers that the Mask R-CNN model overlooked in the chrysotile slide images were detected by the MA-Net model (Fig. 3b).

With regards to accuracy in detecting fibers, the Mask R-CNN model had recall and precision rates of 57% and 46%, respectively, which were not remarkably high; however, the MA-Net model had a high detection accuracy of 95% recall rate and 91% precision rate (Table 2). Generally, in the learning of AI models, instance segmentation models require hundreds to thousands of learning datasets, whereas semantic segmentation models require dozens. In this study, it was inferred that the Mask R-CNN instance segmentation model was not sufficiently trained because only a small number of training datasets were available. Thus, the recall rate was poor. However, both models showed similar results for overcounting, indicating that the poor contour extraction accuracy overcounted fibers that were smaller than the counting criteria by extracting fiber contours larger than they actually were. Therefore, improvement of contour extraction accuracy is needed to reduce overcounting.

**Table 2.** Accuracy of the two artificial intelligence models in detecting fibers.

	Mask R-CNN model	MA-Net model
Recall	57%	95%
Precision	46%	91%

MA-Net, multi-level aggregation network; Mask R-CNN, mask region-based convolutional neural network.

Fiber detection took less than 1 s per image in both AI models. The measurement method using PCM images in Japan takes 25 to 100 min to count fibers in 100 fields of view. The AI models can detect fibers in 100 fields of view within approximately 2 min, making more rapid detection possible. However, this time does not include the time spent manually adjusting the focus and capturing the images. Automating the focus adjustment and image capture is important to increase the usability of this system. One solution is to capture and synthesize multiple images in the depth direction. Although this study was not completed in time, we will consider it in the future. Our results showed that an AI rapid fiber-detection technique—demonstrated in simulated atmospheric samples using PCM image analysis—can detect emission control failures and potential high exposure to asbestos in the demolition and removal of buildings constructed with ACBMs. Therefore, this technique has potential regarding the early detection and correction of atmospheric emissions of asbestos fibers due to inappropriate demolition works.

In this study, we examined the detection of fibers in images of simulated atmospheric samples prepared from asbestos standard samples. Atmospheric samples collected at demolition and removal sites are assumed to contain many particles and non-asbestos fibers. We intend to conduct studies using atmospheric samples collected at actual demolition and removal sites to obtain high accuracy in the AI models’ ability to detect fibers in such samples.

## Conclusion

We studied the use of AI models to detect fibers by analyzing PCM images. Simulated atmospheric samples prepared from amosite and chrysotile standard samples were observed using a PCM, images were captured, and training datasets were created based on the counting results obtained by an expert analyst. The training datasets were used to train an instance segmentation model, namely the Mask R-CNN, and a semantic segmentation model, namely the MA-Net, to detect fibers. Satisfactory results were obtained with the MA-Net model. The overall counting time was reduced to 1/12^th^–1/50th of the time required for counting by an expert analyst.

## Supplementary Material

wxae014_suppl_Supplementary_Material

## Data Availability

The data underlying this article are available in the article and in its online supplementary material.
