# Supplementary material for: Rapid fiber-detection technique by artificial intelligence in phase-contrast microscope images of simulated atmospheric samples
Source: Ann Work Expo Health. 2024 Mar 4;68(4):420–6. doi: 10.1093/annweh/wxae014 (PMC11033560; doi:10.1093/annweh/wxae014)
Supplement: wxae014_suppl_Supplementary_Material [file wxae014_suppl_supplementary_material.pdf]

Supplementary Materials

Rapid Fiber-Detection Technique by Artificial Intelligence in Phase-Contrast Microscope Images of Simulated Atmospheric Samples

Takashi Yamamoto[1)] *, Kazuharu Iwasaki[2)], Yukiko Iida[3)], Ken-ichi Yuki[3)], Fumihiro Nakaji[2)], Hayato Yamashiro[2)], Toshiyuki Toyoguchi[3)], Atsushi Terazono[1)]

[1)] National Institute for Environmental Studies 16-2 Onogawa, Tsukuba, Ibaraki 305-8506 Japan

[2)] Japan NUS Co., Ltd 7-5-25 Nishi-Shinjuku, Shinjuku, Tokyo 160-0023 Japan

[3)] Environmental Control Center Co., Ltd 3-7-23 Sanda-machi, Hachioji, Tokyo 193-0832 Japan

Table S1. Specifications of the computer used.

Item	Specification
CPU	Intel Xeon W-2235 (6 cores and 12 threads)
Memory	32 GB
GPU	NVIDIA GeForce RTX 3090<br>(CUDA cores: 10496; VRAM: 24 GB)
OS	Ubuntu 18.04.5 LTS 64bit

CPU, central processing unit; GPU, graphic processing unit; OS, operating system.

Table S2. Libraries used.

Item	Version	Contents
Anaconda	4.12	Python distribution
Python	3.9.7	Python
PyTorch	1.10.2	Deep learning framework
torchvision	0.11.3	Image analysis framework for image recognition, object detection, segmentation, etc. using PyTorch. Use the source code and trained weight parameter data of the Mask R-CNN model implemented in this library.
segmentation_models_pytorch	0.2.1	Semantic segmentation model library. Used the source code and learned weight parameter data of the MA-Net model implemented in this library.
CUDA Toolkit	11.3	CUDA runtime. Used for model training and inference using PyTorch GPUs.
Albumentations	1.1.0	Image data extension library. Used for data augmentation such as horizontal/vertical inversion of images during learning.
Pillow	8.4.0	Image processing library. Used for image input/output.
OpenCV	4.5.5.64	Image analysis library. Used for creating segmentation masks, extracting contours, detecting circumscribed rectangles considering rotation, and measuring fiber lengths and widths.
NumPy	1.21.2	Multidimensional array math library. Used for segmentation mask post-processing, fiber length and fiber width measurement

MA-Net, multi-level aggregation network; Mask R-CNN, Mask Region-based Convolutional Neural Network.

Table S3. Settings used for the artificial intelligence model learning.

Items	Settings
Image size	Mask R-CNN: 1,600 × 1,200<br>MA-Net: 928 × 928
Data expansion	Horizontal flip, vertical flip, −45° to 45° image rotation, with 50% probability.
Number of mini batches	Mask R-CNN: 2<br>MA-Net: 4
Number of epochs	10
Initial values of weight parameters	Mask R-CNN: Used weight parameter files learned on ImageNet and COCO datasets that come with torchvison.<br>MA-Net: Encoder initial values use weight parameters provided by segmentation_models_pytorch.
Parameter update	Momentum SGD (Stochastic Gradient Descent with Inertia Term)<br>Initial learning rate<br>Mask-RCNN: 0.005<br>MA-Net: 0.0001

MA-Net, multi-level aggregation network; Mask R-CNN, mask region-based convolutional neural network.

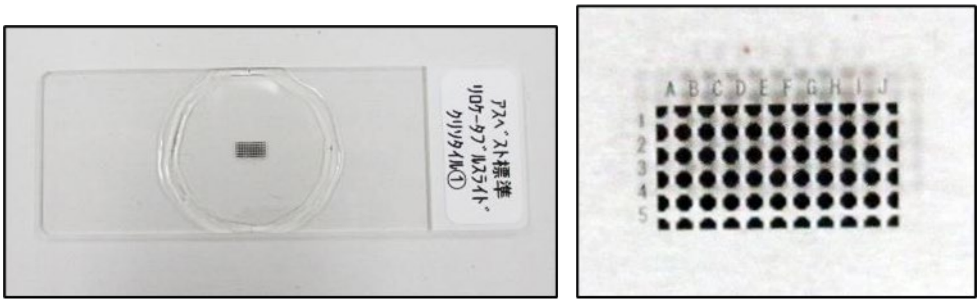

Figure S1. Photographs of a simulated air sample (chrysotile slide). Whole slide (left) and magnified view of the center of the coverslip (right).

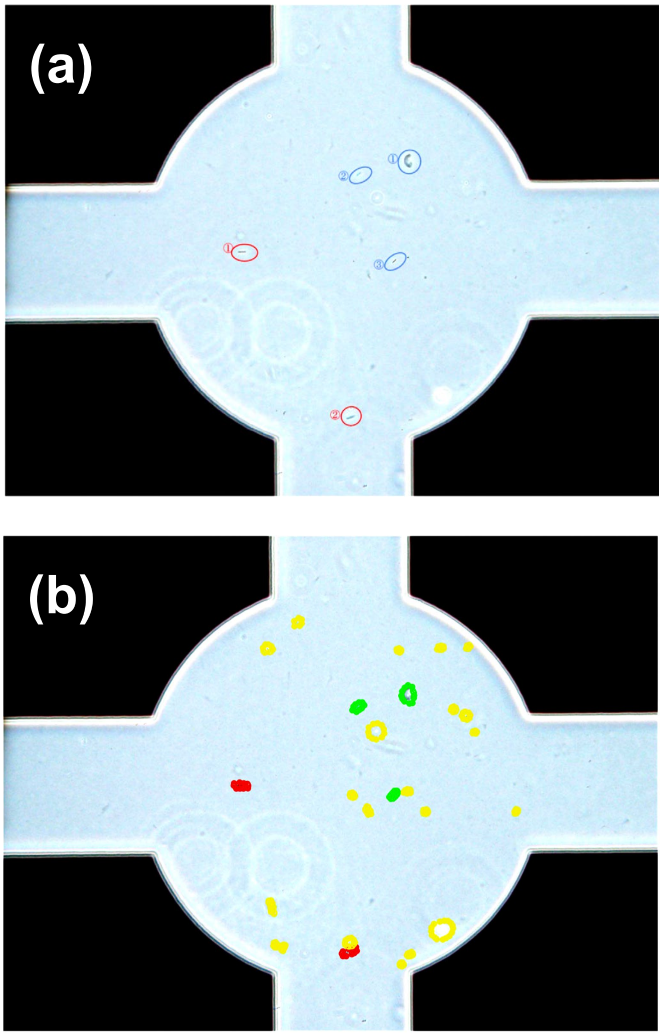

Figure S2. (a) Fiber counting results achieved by an expert analyst and (b) the training datasets created based on those counting results.

In (a), fibers that meet the fiber counting criteria are circled in red and those that do not are circled in blue. In (b), the contours of fibers that meet the fiber counting criteria (red), fibers that do not (green), and other particles (yellow) were extracted to create training datasets.

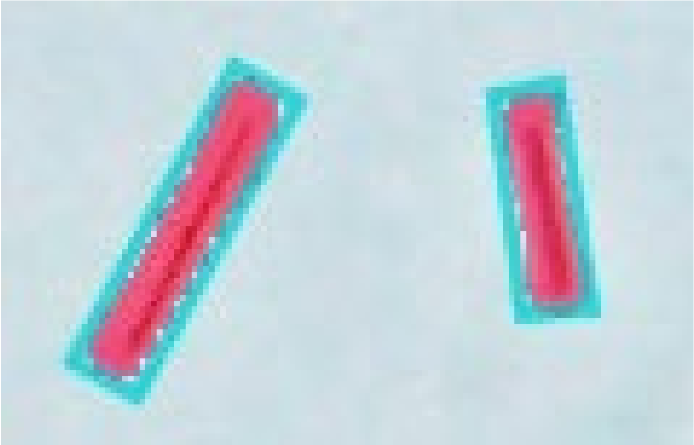

Figure S3. The segmentation mask (red) and bounding rectangle that considers rotation (light blue).

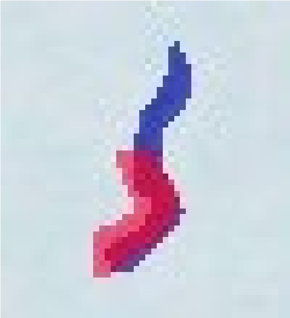

Figure S4. The contours of a fiber in the Mask R-CNN model's evaluation data (blue) and predicted data (red).

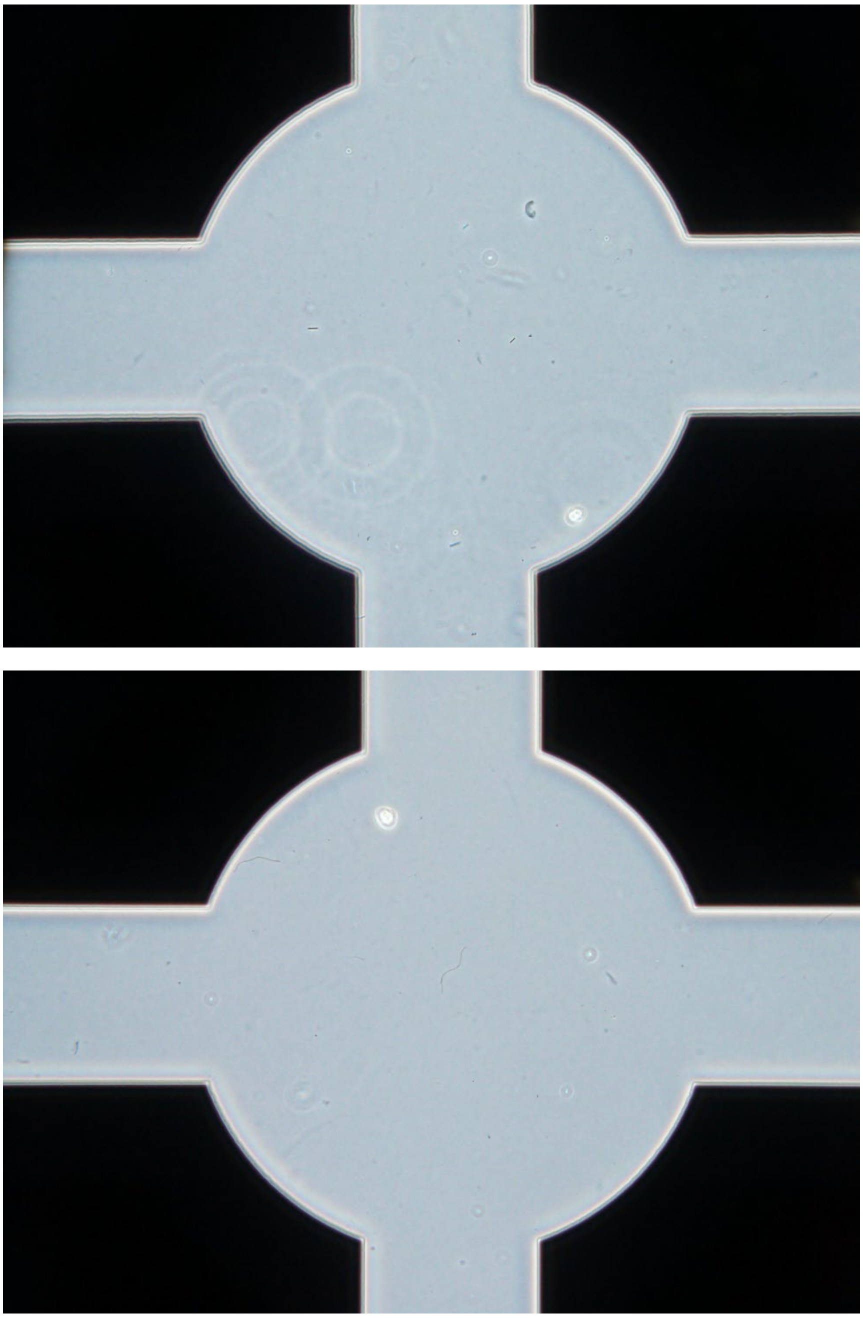

Figure S5. Original images of fields of view of asbestos slides.

Upper image is of amosite slide (Amo-1-B1) and lower image is of chrysotile (Chr-1-D2) slide.
